# Comparison of efficacy and safety of Chinese patent medicine in the treatment of postmenopausal osteoporosis: A protocol for systematic review and network meta-analysis

**DOI:** 10.1097/MD.0000000000022734

**Published:** 2020-10-16

**Authors:** Hongqiang An, Jifeng Zhao, Jiahao Wang, Chuancheng Li, Zhenyuan Jiang, Junpeng Yao, Xiao Zhang, Jianlin Wu

**Affiliations:** a Shandong University of Traditional Chinese Medicine; b Xintai People's Hospital; c Jinan city Hospital of Integrated Traditional Chinese and Western Medicine, PR China.

**Keywords:** Chinese patent drugs, network meta-analysis, osteoporosis, postmenopausal, protocol

## Abstract

**Background:**

Postmenopausal osteoporosis (PMOP) is the focus and difficult problem in the world at present, and we found that Chinese patent medicine(CPM) shown a more miraculous effect. Many kinds of Chinese patent medicine have been proved to be effective in the treatment of this disease, but it is still unclear which kind of Chinese patent medicine has the best effect. Therefore, we propose a network meta-analysis (NMA) protocol to observe the efficacy of various CPM for this disease and provide guidance for clinical practice.

**Methods:**

We will use the NMA method to complete this study. First, all the randomized controlled trials of CPM or CPM combined with western medicine in the treatment of PMOP were collected by searching all online Chinese and English databases. The information time limit is from the establishment of the database to August 30, 2020. Then 2 staff members will sift through all the literature and analyze the data using Stata and Winbugs.

**Results:**

Through this analysis, we will observe and rank the clinical effects of different CPM for PMOP. The main evaluation indexes include: New fracture, Quality of life, Severe side effects, Death from all causes. Secondary outcome indicators include Bone Mineral density, clinical efficiency, and some laboratory indicators, such as estradiol, serum calcium, serum, etc.

**Conclusion:**

This study will rank the therapeutic effects of various proprietary Chinese medicines in the treatment of PMOP, which will be helpful in improving the PMOP treatment regimen.

INPLASY registration number: INPLASY202090047.

## 1 Introduction

As a kind of primary osteoporosis, postmenopausal osteoporosis (PMOP) is caused by the decrease of ovarian function and the rapid decrease of plasma estrogen level in postmenopausal women, which leads to the decrease of bone mass, thus leading to the rapid increase of the incidence of osteoporosis.^[1]^ PMOP is a major health risk for older persons. It often results in high morbidity and mortality rates for brittle fractures, as well as increased socio-economic costs.^[2–4]^ With the advent of aging society and peoples life expectancy, the cost of fracture care caused by osteoporosis will increase the medical burden of the whole society.^[5–7]^ According to statistics, there are 10 million cases of bone fracture caused by osteoporosis worldwide every year, and two-thirds of them are women.^[8]^ Organizations such as the IOF are also studying the epidemiology of osteoporosis and fractures. Research now suggests that people with high rates of osteoporosis are moving from Europe to Asia. The number of fractures in elderly patients in Asia is expected to increase eightfold between 2000 and 2050. In addition, 50% of hip fractures worldwide occur in Asia.^[9]^ Therefore, the prevention and treatment of PMOP to reduce the occurrence of brittle fractures and reduce the socio-economic burden has been an important research topic. Currently, there are many drug treatments for PMOP, mainly including basic supplements (bone mineralization drugs, such as calcium and vitamin D), bone resorption inhibitors, bone formation promoters, other mechanism drugs, and Traditional Chinese medicines.^[10,11]^ On the other hand, there are problems with all kinds of treatments. For example, bisphosphonates, which inhibit osteoclast mediated bone resorption, are currently considered to be the first line of treatment for osteoporosis.^[12]^ However, both it and Denosumab were associated with osteonecrosis of the mandible and an atypical femoral shaft fracture.^[13–15]^ Cathepsin K inhibitors, which reduce bone resorption, reduce the risk of fractures, but they also increase the risk of cardiovascular disease.^[16]^ The study found that oral calcitonin did not reduce the risk of fracture.^[17]^ At present, there are also many literatures on the treatment of PMOP with Chinese patent medicine(CPM).^[18–22]^ Data showed that Xianling Gubao had a good therapeutic effect on osteoporosis in rats, and there was no obvious toxicity or adverse reactions when the dose exceeded 6 times of the daily recommended dose.^[21]^ Yigu capsule can promote bone formation, inhibit bone absorption and increase sex hormone level.^[23]^

Gu Shukang capsule has the protective effect of promoting bone formation and preventing the apoptosis of bone cells 18.^[18]^ However, a review of all the current studies found that there was a lack of a systematic analysis of the therapeutic effects and safety of various Chinese patent medicine, and there was still no research to rank their therapeutic effects. Based on these findings, we conducted this study on the treatment of osteoporosis by Chinese patent medicine and proposed a NMA scheme to discuss the efficacy of various drugs.

## 2 Methods

In this study, we will adopt the NMA and carry out literature review according to PRISMA statements.^[24]^

### 2.1 Study registration

This NMA has been registered on the INPLASY. The registration number is INPLASY202090047(URL= https://inplasy.com/inplasy-2020-9-0047/).

### 2.2 Inclusion criteria

#### 2.2.1 Type of study

We will include published randomized controlled trials (RCTS) in China and internationally. Whether blind or not, and language is limited to Chinese and English.

#### 2.2.2 Participants

According to the PMOP Diagnostic Criteria 21 issued by the American Association of Clinical Endocrinologists (AACE),^[25]^ we will include patients diagnosed with PMOP, regardless of age, race, ethnicity, primary disease, or Clinical stage.

#### 2.2.3 Interventions and comparators

Intervention measures: The treatment group was treated with a CPM or CPM combined with conventional western medicine (CWM). The control group was treated with CWM only. CWM must be the same between groups in the same study and used as a base intervention. Routine therapy includes guidelines recommending drugs such as bisphosphonates, hormones, calcitonin, and basic supplements for bone health such as calcium and vitamin D. There is no limit on dosage, usage, and course of treatment. The interventions in both groups did not include intravenous medication, herbal decoction, or other extrinsic methods such as acupuncture and massage.

#### 2.2.4 Outcomes

Main outcome indicators: new fracture; Quality of life; Severe side effects; Death caused directly or indirectly.

Secondary outcome indicators: bone mineral density (BMD), estradiol, serum calcium, serum phosphorus, serum alkaline phosphatase, bone glaprotein, tartrate-resistant acid phosphatase, bone alkaline phosphatase, pain degree, Clinical efficiency.

### 2.3 Exclusion criteria

Cross-sectional studies, studies without control group, animal experiments and reviews; Literature whose data cannot be extracted or whose experimental design is not rigorous. The original materials were not publicly published documents; Duplicate or plagiarized literature.

### 2.4 Search strategy

We will use computers to search Embase, Pubmed, Cochrane Library, Web of Science, CNKI, WanFang, VIP, CBM databases, and the ClinicalTrials.gov clinical registration system to collect RCT for PMOP. The retrieval time is limited to August 30, 2020 from the beginning of repository construction. The retrieval method will adopt the combination of subject words and free words. Take PubMed as an example, and the retrieval strategy is shown in Table 1.

**Table 1:**
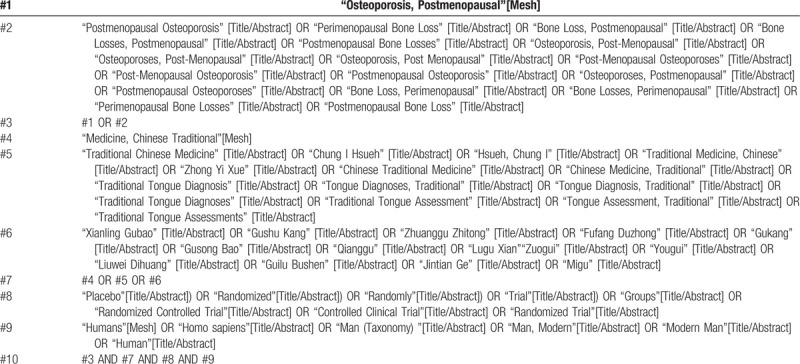
Search strategy for PubMed.

### 2.5 Study selection and data extraction

In this study, two researchers will independently screen the literature, extract the data and cross-check them according to the pre-determined screening criteria. In case of disagreement, it may be decided by discussion or by a third party. EndNote literature management software will be used to review the imported literature, and a preliminary screening will be conducted by reading the title and abstract of the literature, so as to exclude the literatures that obviously do not meet the inclusion criteria. References that may meet the inclusion criteria will be further read and a second screening will be conducted to determine eventual inclusion. If necessary, we will contact the author of the original study by telephone or email to obtain important information for this study. Excel data extraction table will be established to extract data, including: General research data, such as article title, first author, year of publication; Basic information of subjects, such as diagnosis, age, source of cases, course of disease, etc; Key contents of bias risk assessment; Intervention measures and control measures; Main research indicators.

### 2.6 Risk of bias assessment

We will evaluate the quality of the included literature using the bias risk assessment tool recommended by Cochrane System Reviewers’ Manual 5.3.^[26]^ Including: Random method; Allocation hiding; Blind the researchers and participants; Blind the research results; The integrity of the result data; Selective reporting of research results; Whether there are other sources of bias. Each evaluation result will be divided into low risk of bias, high risk of bias and unclear.

### 2.7 Statistical analysis

Counting data will adopt odds ratio (OR), and continuous variable data will adopt either mean difference (MD) or standardized mean difference (SMD). As treatment effects, both are represented as the effect value and its 95% Confidence interval (CI). RevMan 5.3 software will be used for bias evaluation, and Stata 16.0 software will be used for heterogeneity analysis and evidence diagrams for NMA.

We will determine the size of heterogeneity quantitatively by *I*^2^. When *I*^2^ < 50% and *P* > .1, we will assume that there is no statistical heterogeneity in each study, and we will use the fixed-effect model for Meta-analysis. When *I*^2^ ≥ 50% and *P* ≤ .1, statistical heterogeneity among studies will be considered. At this time, the source of heterogeneity needs to be further analyzed. After excluding the clinical heterogeneity factors, the random-effect model is used for Meta-analysis. If clinical heterogeneity exists, we will use subgroup analysis and meta-regression analysis. If the source of heterogeneity is unknown, meta-analysis is abandoned and descriptive analysis is adopted. Sensitivity analysis will be used to determine the robustness of the results.

Therefore, when running win-Bugs, random effects models will be used for analysis. The number of iterations will be set to 100,000. The first 5000 iterations will be used to anneal to eliminate the influence of the initial value, and then the iteration history diagram will be drawn to evaluate the convergence degree of the model. When there is a closed loop in the random effects model, the consistency between direct comparison and indirect comparison is determined by the inconsistency factor (IF value). When the IF value 95%CI starts at 0, this means that the direct evidence is consistent with the indirect evidence. At last, the evidence of small sample effect in the network is identified by drawing “comparation-correction” funnel plot.

#### 2.7.1 Grading the quality of evidence

Grades of Recommendations Assessment, Development and Evaluation (GRADE) will be used to evaluate the quality of evidence. We’ll evaluate from the following 5 areas, including: Risk of bias, Indirectness, Inconsistency, imprecision, and publication bias^[27]^.

#### 2.7.2 Ethics and dissemination

This study does not involve personal and human trial data and therefore does not require ethical approval.

## 3 Discussion

The incidence of PMOP is the result of multi-factors, multi-links and multi-targets, which seriously affects the health of postmenopausal women. As the representative of compound composition therapy, Traditional Chinese medicine has the advantage of multi-target effect in the treatment of osteoporosis. Chinese patent medicines commonly used at present are made from herbal medicines and approved by the State Medical Products Administration. It generally has pills, tablets, capsules, granules and other dosage forms, and it has the characteristics of safety, convenience, easy to promote. In this study, we hope to evaluate the advantages, disadvantages, and efficacy of various drugs, so as to help relieve patients’ pain and provide guidance for clinical treatment. However, the results of our analysis may be biased due to data inequality. Therefore, in the future work, we still need to pay attention to these research progress. Multi-center clinical studies and evidence will be used to evaluate the efficacy and safety of CPM for PMOP.

## Author contributions

**Conceptualization:** Hongqiang An.

**Data curation:** Hongqiang An, Jianlin Wu.

**Formal analysis:** Hongqiang An.

**Funding acquisition:** Jianlin Wu.

**Investigation:** Hongqiang An.

**Methodology:** Hongqiang An.

**Project administration:** Hongqiang An.

**Resources:** Hongqiang An, Jiahao Wang, Xiao Zhang.

**Software:** Jiahao Wang, Chuancheng Li, Zhenyuan Jiang, Junpeng Yao, Xiao Zhang.

**Validation:** Hongqiang An.

**Visualization:** Hongqiang An.

**Writing – original draft:** Hongqiang An, Jifeng Zhao.

**Writing – review & editing:** Hongqiang An, Jianlin Wu.
